# Regional disparities in medical equipment distribution in the Slovak Republic – a platform for a health policy regulatory mechanism

**DOI:** 10.1186/s13561-017-0176-0

**Published:** 2017-11-09

**Authors:** Beáta Gavurová, Viliam Kováč, Ján Fedačko

**Affiliations:** 1 Department of Banking and Investment, Faculty of Economics, Technical University of Košice, Němcovej 32, 04001 Košice, Slovak Republic; 2 Department of Finance, Faculty of Economics, Technical University of Košice, Němcovej 32, 04001 Košice, Slovak Republic; 3 1st Department of Internal Medicine, Louis Pasteur University Hospital in Košice, Trieda Slovenského národného povstania 1, 04011 Košice, Slovak Republic; 4 Centre of Excellence in Atherosclerosis Research, Pavol Jozef Šafárik University in Košice, Trieda Slovenského národného povstania 1, 04011 Košice, Slovak Republic

**Keywords:** Medical equipment, Healthcare, Health facility, Region, Regional disparity

## Abstract

**Background:**

This study aims to examine the localisation of selected parameters in the deployment and use of medical equipment in the Slovak Republic and to verify potential regional disparities. The study evaluates the benefits of an analytical platform for regulatory mechanisms in the healthcare system.

**Methods:**

The correspondence analysis is applied to the entire data set containing information regarding medical equipment distribution and mortality.

**Results:**

The results highlight regional differences in the use of medical equipment throughout the analysed period from 2008 to 2014. The total amount of medical equipment increased slightly to 9192 devices during the time span. In 2014, there was a significant decrease of 16.44%. Disparities are found in the frequencies and structure of medical equipment. In some regions, medical equipment is not present or is present in low numbers.

**Conclusions:**

The results regarding regional disparities demonstrate the regional development of the amount of medical equipment. The deployment of medical equipment is not proportional, and not all of the analysed devices are available in each region. The tests also indicate the appropriateness of the amount of medical equipment and create a platform for further investigation. The results of the analysis suggest the unsuitable distribution of medical equipment throughout the Slovak regions, where there are significant regional disparities. These findings can serve as a monitoring platform to evaluate the accessibility and efficiency of medical equipment usage.

**Trial registration:**

No human participants were involved in the research.

## Background

In recent decades, the field of medical equipment and the promotion of medical and economic potential have received attention in terms of detecting the increase in the efficiency of health systems as well as rapid progress in the development and implementation of new knowledge from science and research. New medical equipment must be evaluated before it is introduced. This process is called health technology assessment. This multidisciplinary process summarises information regarding the medical, economic, social and ethical issues associated with the use of particular medical equipment. Health technology assessment has a specific position in scientific research [1–3]. It creates a link between science and decisions in health policy [4–6]. As evidenced by the International Monetary Fund studies comparing the effectiveness of health technology, this process has been ongoing in Europe for approximately a decade [7]. The Slovak Republic possesses great potential for eliminating inefficiencies [8] and determining the efficient allocation of financial resources for the health system. In some countries, health technology assessment involves a measurement process for safety and efficiency [9]. The Slovak Republic significantly lags behind in the implementation process. The process of evaluating medical equipment directly relates to the many regulatory processes in the health system sector that are built on the pillars of the existence, acceptability, accessibility and quality of healthcare [10]. These aspects are determined by the sustainability of healthcare costs, which is a priority in the creation of a state budget. Transparent regulatory processes in healthcare along with product innovations and services and increased efficiency can greatly eliminate the disparity between resources and the cost of healthcare in a country. Studies have examined possibilities for increasing productivity in the interest of sustainable healthcare costs at optimal limits [11, 12]. Within the European Union as well as the Organisation for Economic Co-operation and Development, significant differences in the availability of resources for mental healthcare are evident. Over the past three decades, the most common causes of death changed from infectious diseases to chronic diseases, putting further pressure on the efficiency and effectiveness of healthcare [13, 14].

Management of spending on the healthcare system became a major health policy goal after the global financial crisis in 2008. Although these expenditures turned out to be stabilised after 2010, they remained considerably below the European average. There are several health indicators that can serve as evaluating points. The health care system in the Slovak Republic is based on universal coverage. It means health insurance is compulsory for each individual. General coverage is provided by basic benefit package. On the other hand, a competitive insurance model with selective contracting of health care providers by health insurers and flexible pricing of health services is offered too. After fulfilling certain explicit criteria, there are no barriers to entry the healthcare provision market or the health insurance market. Generally, healthcare is provided free of charge. It is paid by the health insurers and also some additional payments are delivered by them.

There are three health insurance companies which compete for clients based on the quality and variety of their contracted services. They are obliged to ensure accessible healthcare regulated by legislation – this means to contract a sufficient network of providers as determined by the Ministry of Health. The Health Care Surveillance Authority (Úrad pre dohľad nad zdravotnou starostlivosťou) is responsible for surveillance over the health insurance and healthcare provision. Since 2005, all the health insurance companies are joint stock companies. There is one state-owned health insurer and two privately owned health insurance companies. The first one possess roughly a 65-per-cent share of the market.

The Slovak healthcare system is now in a process of adopting new strategic planning framework. Firstly, it was introduced by the government in July 2014. This framework aims to ensure integrated outpatient care, to contain overutilization, and to restructure inpatient health care. In 2014, total expenditure in the health system was 8.1% of the gross domestic product. This figure is still significantly lower than the European Union average of 9.5%. Public resources brought 72.5% of total expenditure in the health system in that year – slightly lower than the European Union average of 76.2%. The main source of revenue of the health system is represented contributions from employees and employers, self-employed, voluntarily unemployed, publicly financed contributions on behalf of economically inactive persons and dividends. Compulsory health insurance contributions are collected by the health insurance companies. The main issue in field of financing is continually rising substantial debt of the healthcare facilities. On the other hand, the sole investments come only from the European Union structural funds [15].

From a demographic point of view, the situation in Europe is not appropriate in its current state. According to forecasts, in 2030, up to 30% of the European population will be over 65 years old, and fewer people will be in the age range for economic activity – over 15 and under 65 [16, 17]. This situation is caused by the projected decrease from 67% to 56% in the share of the population in the economically active age range. These reported demographic changes will have a significant impact on public finances in the European Union and will put pressure on health policies. It is important to give consideration to public spending linked to the issue of age, such as pensions, health and long-term care. These expenditures are expected to grow by 4.1% of the gross domestic product in 2060 compared to 2010. This represents an increase of approximately 25% to 29% of the gross domestic product. For expenditures on pensions, an increase from 11.3% to approximately 13% of the gross domestic product is expected. There will be significant differences determined by the structure and methods of implementation of potential pension reforms among the European countries. Because there are large discrepancies in the resources available for healthcare, the extent of differences and the rate of diffusion of new medical practices and equipment will play an important role. These aspects are optimally evaluated by a particular measurement system, avoidable mortality, which is the subject of development and review by many research teams around the world [8, 18–23]. This measurement system considers the extent of the implementation of modern healthcare technologies in the context of interventions that are the basis for recovery and the inclusion or exclusion of individual diagnoses of avoidable mortality. It is sometimes called treatable or preventable mortality. Negative values for avoidable mortality in a country may also reflect the significant non-availability of appropriate medical equipment, poor quality of provided healthcare services, or a combination of both. From a macroeconomic perspective, the smallest volume 872.9 USD of financial resources in healthcare per capita is spent in Romania, where the standardised mortality rate of 304 per 100,000 inhabitants is the highest among the European Union members. In contrast, Luxembourg is characterised by the highest amount 6340.6 USD, and its standardised mortality rate of 89 per 100,000 inhabitants is one of the lowest among the surveyed countries. The lowest treatable mortality rates are found in Denmark, Sweden, the Netherlands, Spain, and France. There are minimal differences in the values of treatable mortality among these countries; the significant differences are in the volume of expenditures on their national health systems per capita – for instance, Spain’s expenditure is 3144.9 USD [10].

These facts prompted us to explore the situation in a field related to mortality in the Slovak Republic. Usage of the medical equipment influences mortality rate in general. Therefore, the primary aim of this study is to examine the localisation of the selected parameters in the deployment and use of selected medical equipment in the Slovak Republic and to verify the potential regional disparities. This study also evaluates the benefits of a quantitative analytical platform for regulatory and stabilising mechanisms in the health system. The unordinary methodical approaches are able to reveal the desired objective – a comprehensive evaluation of the observed equipment. It is not only to pick up advantages or disadvantages, but also to prepare a platform for further research. That is why, not multifaceted outputs are shown in the analysis, but rather significant points are addressed to be investigated in the subsequent steps. The study also assesses a position of an analytical platform in field of the healthcare system not only for the running regulatory mechanisms, but also to prepare new types of regulatory mechanisms.

## Methods

The data is provided by the National Health Information Center (Národné centrum zdravotníckych informácií). The dataset involves numbers of the individual type of medical equipment in the particular healthcare facilities. There are twenty-nine medical equipment types included in this analysis. Their labels in the subsequent tables and figures are as follows: angiograph – 1; brachytherapy apparatus – 2; bronchoscope – 3; cystoscope – 4; dialysis monitor – 5; electrocardiograph – 6; electroencephalograph – 7; electromyograph – 8; endoscope – 9; laparoscope, arthroscope – 10; gamma camera – 11; gastroscope, duodenoscope – 12; isotope irradiator – 13; colonoscope, sigmoidoscope, proctoscope – 14; colposcope – 15; cryogenic device – 16; laryngoscope – 17; laser – 18; linear accelerator – 19; lithotripter – 20; magnetic resonance imaging device – 21; mammograph – 22; monitoring device – 23; positron tomograph – 24; x-ray – 25; tomograph – 26; ultraviolet and infrared emitter – 27; ultrasound device – 28; uretroscope – 29; high-frequency device – 30.

Regional disparities are observed according to the third level of the nomenclature of territorial units for statistics. This administrative level of the territorial division of the Slovak Republic represents the highest tier of the administrative division of the country. This division is applied from the perspective of self-government because these self-governing regions also manage the health facilities in their territory.

There are eight self-governing regions in the Slovak Republic, which have the following labels in the subsequent tables and figures: the Banská Bystrica Self-governing Region – BC; the Bratislava Self-governing Region – BL; the Košice Self-governing Region – KI; the Nitra Self-governing Region – NI; the Prešov Self-governing Region – PV; the Trenčín Self-governing Region – TC; the Trnava Self-governing Region – TA; the Žilina Self-governing Region – ZI.

Several mathematical relations are applied in the analytical part of the study. They are mentioned in their applied forms for the purposes of this analysis. The majority of the analysis is conducted in the form of a correspondence analysis. The rest of the analysis involves identifying similarities between the types of medical equipment. All the analytical outputs are executed in the statistical software environment R using among others the package vcd handling visualising categorical data.

The equipment profile is calculated as follows [24]:

1 $$ {EP}_{e;r}=\frac{n_{e;r}}{\sum \limits_{r=1}^c{n}_{e;r}}, $$

where the variables are as follows:

- *EP*_*e; r*_ – equipment profile of the e equipment type in the r self-governing region;
- *e* – the equipment type;
- *r* – the self-governing region;
- *n*_*e; r*_ – number of the e equipment type in the r self-governing region;
- *c* – number of the self-governing regions.

The quantification of the regional equipment profile is performed in the following way [24]:

2 $$ {REP}_r=\frac{\sum \limits_{e=1}^t{n}_{e;r}}{\sum \limits_{e=1}^t\sum \limits_{r=1}^c{n}_{e;r}}, $$

where the variables are as follows:

- *REP*_*r*_ – regional equipment profile of the r self-governing region;
- *t* – number of the equipment types;
- *e* – the equipment type;
- *r* – the self-governing region;
- *n*_*e; r*_ – number of the e equipment type in the r self-governing region;
- *c* – number of the self-governing regions.

There are 8 regions in the Slovak Republic, which is why *c* is equal to 8. In our case, there are 29 types of equipment, so *t* is equal to 29. To compute the ratio of the amount of equipment to the number of inhabitants, the population as of 31 December 2014 is used.

The chi-square distance is calculated as follows [25]:

3 $$ {D}_{e_1;{e}_2}=\frac{EP_{e_1;r}-{EP}_{e_2;r}}{REP_r}, $$

where the variables are as follows:

- *e*_*1*_ – the equipment type;
- *e*_*2*_ – the equipment type;
- ${D}_{e_1;{e}_2}$ – regional equipment profile of the *r* self-governing region;
- *r* – the self-governing region;
- ${EP}_{e_1;r}$ – equipment profile of the *e*_*1*_ equipment type in the *r* self-governing region;
- ${EP}_{e_2;r}$ – equipment profile of the *e*_*2*_ equipment type in the *r* self-governing region;
- *REP*_*r*_ – regional equipment profile of the *r* self-governing region.

The output of the analysis is demonstrated on the plots which visualise localisation of the observed types of the medical equipment too. The applied types of the diagrams are mosaic plot and association plot. The first one introduces a multidimensional graphical method to envision the data from several qualitative variables. This method represents visualisation of contingency table involving discrete values in general. A standard way is to demonstrate two-dimensional contingency table. Dependence between parts of mosaic plot is found horizontally and vertically too. One reliance represents a proportion among categories of the particular variable, whilst the other one relation among the variables of the same category. Each cell pictured by rectangle created by rectangle exhibits a proportion of the particular category for the particular variable. Appropriate dimension of rectangle is determined by its share of the total for the variable [26, 27]. This type of diagram is also called mekko chart.

On the other hand, the association plot represents another way of visualisation of two-dimensional contingency table. Every cell visualised like rectangle represents one category of the individual variable. All the cells for each category and each variable are localised relative to a baseline representing an independent state. On the one hand, a case with observed frequency higher than expected frequency is demonstrated by cell rising above the line. On the other hand, a case, which shows smaller observed frequency than is expected, falls below the line. Comparison of observed and expected frequency is measured under the null hypothesis of independence [28, 29]. Sometimes, this type of diagram is referred to as Cohen-Friendly association plot.

## Results

**Fig. 1:**
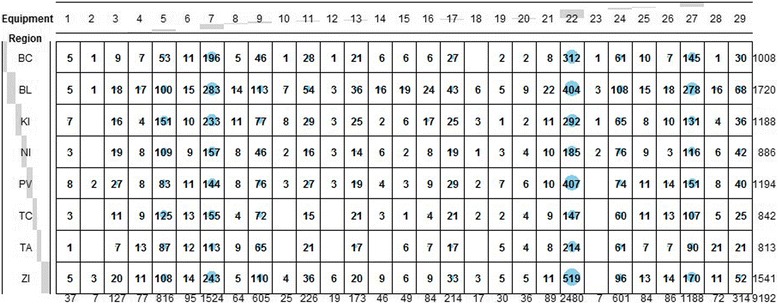
Equipment distribution according to the self-governing region and its type

An initial look at the dataset demonstrates the localisation of the individual types of equipment in the particular self-governing regions in Fig.1.

The localisation of the equipment types in the separate self-governing regions is demonstrated in Fig. 1. The x-axis shows the order of all the equipment types recognised by the dataset from the National Centre of Health Information of the Slovak Republic, and the y-axis represents the administrative division of the Slovak Republic at the level of the territory units – the self-governing regions. The shading of the x-axis tags represents the share of the amount of equipment of that type to the total number of equipment of all 29 types on the x-axis and the share of the equipment located in the particular self-governing regions to the total amount of the equipment in the entire Slovak Republic on the y-axis. The size of the rings in the individual cells and their shading are determined by the chi-square distance. The larger the ring is, the larger the distance is between the given rows. This indicates that these types of equipment are more dissimilar. We have chosen this visualisation because more equipment types are similar rather than different. To analyse the distribution of the devices throughout the Slovak Republic, we quantify an equipment profile for each type of equipment in 2008 and in 2014. This profile presents the share of that type of equipment localised in the particular self-governing region to the total amount of equipment in the entire country. The equipment profiles in 2008 are shown in Table 1.

**Table 1:** Equipment profiles in 2008

Profile	BC	BL	KI	NI	PV	TC	TA	ZI
1	0.125	0.2	0.225	0.025	0.175	0.075	0	0.175
2	0.1818	0.0909	0.3636	0	0	0.0909	0	0.2727
3	0.0922	0.0922	0.1844	0.156	0.1773	0.078	0.0567	0.1631
4	0.0532	0.2234	0.1596	0.117	0.0957	0.1064	0.1064	0.1383
5	0.0669	0.6642	0.0291	0.0436	0.0392	0.0218	0.0567	0.0785
6	0.1342	0.2067	0.1902	0.0797	0.084	0.0926	0.0632	0.1493
7	0.1827	0.1731	0.1154	0.0865	0.1058	0.1154	0.1154	0.1058
8	0.1304	0.2174	0.2174	0.1449	0.1159	0.029	0.0435	0.1014
9	0.1084	0.1756	0.1979	0.0912	0.1188	0.0706	0.0826	0.1549
10	0.0909	0.2955	0.2273	0.0455	0.2273	0	0.0455	0.0682
11	0.1348	0.2348	0.187	0.0609	0.0652	0.0522	0.0739	0.1913
12	0.1481	0.1111	0.2222	0.1481	0.0741	0.037	0	0.2593
13	0.1045	0.204	0.194	0.0995	0.1045	0.0995	0.0796	0.1144
14	0.1136	0.3636	0.1364	0.0682	0.0455	0.0682	0	0.2045
15	0.0625	0.25	0.25	0.0625	0.0625	0.0312	0.1562	0.125
16	0.0909	0.2597	0.2468	0.0779	0.0779	0.026	0.1039	0.1169
17	0.1923	0.2564	0.1496	0.0684	0.1026	0.0556	0.0513	0.1239
18	0.1429	0.3571	0.1429	0	0.0714	0.1429	0	0.1429
19	0.1212	0.1818	0.1212	0.1515	0.1212	0.0606	0.1212	0.1212
20	0.1212	0.2121	0.303	0.1515	0.1515	0	0.0303	0.0303
21	0.0972	0.2639	0.1806	0.0833	0.125	0.0694	0.0694	0.1111
22	0.1275	0.2064	0.1779	0.0834	0.1209	0.0491	0.0615	0.1733
23	0	0.6667	0	0.3333	0	0	0	0
24	0.1212	0.1812	0.15	0.1288	0.1038	0.0762	0.0938	0.145
25	0.1714	0.2	0.1286	0.0857	0.1143	0.0571	0.0857	0.1571
26	0.0333	0.2	0.0667	0.1333	0.2333	0.1667	0	0.1667
27	0.1164	0.2578	0.1362	0.1185	0.105	0.0759	0.0613	0.1289
28	0.0448	0.2687	0.1642	0.0746	0.1045	0.0448	0.1791	0.1194
29	0.0989	0.1943	0.2792	0.0777	0.0954	0.0742	0.0424	0.1378

The unsuitable localisation of the devices among the self-governing regions in 2008 is seen mainly in the case of the least numerous types that are mentioned above in Table 1. Two exceptions appeared. The first one is the colposcope, which has 44 pieces and is the 18th most numerous device; however, 16 of them – 36.36% – are localised in one self-governing region. The second one is the laryngoscope, the 14th most numerous type of equipment. Its total number of 77 includes a 25.97% share and a 24.68% share in the two most abundant regions and zero in one region. The state at the end of the observed period is shown in Table 2.

**Table 2:** Equipment profiles in 2014

Profile	BC	BL	KI	NI	PV	TC	TA	ZI
1	0.1351	0.1351	0.1892	0.0811	0.2162	0.0811	0.027	0.1351
2	0.1429	0.1429	0	0	0.2857	0	0	0.4286
3	0.0709	0.1417	0.126	0.1496	0.2126	0.0866	0.0551	0.1575
4	0.0909	0.2208	0.0519	0.1039	0.1039	0.1169	0.1688	0.1429
5	0.065	0.1225	0.185	0.1336	0.1017	0.1532	0.1066	0.1324
6	0.1286	0.1857	0.1529	0.103	0.0945	0.1017	0.0741	0.1594
7	0.1158	0.1579	0.1053	0.0947	0.1158	0.1368	0.1263	0.1474
8	0.0781	0.2188	0.1719	0.125	0.125	0.0625	0.1406	0.0781
9	0.076	0.1868	0.1273	0.076	0.1256	0.119	0.1074	0.1818
10	0.04	0.28	0.32	0.08	0.12	0	0	0.16
11	0.1239	0.2389	0.1283	0.0708	0.1195	0.0664	0.0929	0.1593
12	0.0526	0.1579	0.1579	0.1579	0.1579	0	0	0.3158
13	0.1214	0.2081	0.1445	0.0809	0.1098	0.1214	0.0983	0.1156
14	0.1304	0.3478	0.0435	0.1304	0.087	0.0652	0	0.1957
15	0.1224	0.3878	0.1224	0.0408	0.0612	0.0204	0.1224	0.1224
16	0.0714	0.2857	0.2024	0.0952	0.1071	0.0476	0.0833	0.1071
17	0.1262	0.2009	0.1168	0.0888	0.1355	0.0981	0.0794	0.1542
18	0	0.3529	0.1765	0.0588	0.1176	0.1176	0	0.1765
19	0.0667	0.1667	0.0333	0.1	0.2333	0.0667	0.1667	0.1667
20	0.0556	0.25	0.0556	0.1111	0.1667	0.1111	0.1111	0.1389
21	0.0899	0.2472	0.1236	0.1124	0.1124	0.1011	0.0899	0.1236
22	0.1258	0.1629	0.1177	0.0746	0.1641	0.0593	0.0863	0.2093
23	0.1429	0.4286	0.1429	0.2857	0	0	0	0
24	0.1015	0.1797	0.1082	0.1265	0.1231	0.0998	0.1015	0.1597
25	0.119	0.1786	0.0952	0.1071	0.131	0.131	0.0833	0.1548
26	0.0814	0.2093	0.1163	0.0349	0.1628	0.1512	0.0814	0.1628
27	0.1221	0.234	0.1103	0.0976	0.1271	0.0901	0.0758	0.1431
28	0.0139	0.2222	0.0556	0.0833	0.1111	0.0694	0.2917	0.1528
29	0.0955	0.2166	0.1146	0.1338	0.1274	0.0796	0.0669	0.1656

The equipment profiles perform very similarly in 2014 in comparison to 2008, as seen in Table 2. The colposcope is still an instance of unsuitably located equipment, although with the increase of its total number to 46, its share in the same self-governing region fell to 34.78%. The share of laryngoscopes in the most abundant region increased to 28.57%, and the zero number in another region disappeared.

To examine the profile of the self-governing regions from with regard to equipment type, a regional equipment profile is applied. It characterises the particular type of medical device in light of all the self-governing regions. The regional equipment profile expresses the mean share of all the equipment profiles in each region, as shown in Table 3.

**Table 3:** Regional equipment profiles

Year	BC	BL	KI	NI	PV	TC	TA	ZI
2008	0.119	0.2458	0.1658	0.0894	0.1023	0.0649	0.0672	0.1457
2009	0.1246	0.2118	0.1756	0.0875	0.1107	0.0719	0.0647	0.1533
2010	0.1178	0.2043	0.1939	0.0828	0.1092	0.0751	0.0696	0.1473
2011	0.117	0.2024	0.1978	0.0798	0.1091	0.0744	0.0684	0.151
2012	0.1187	0.2011	0.1956	0.0818	0.1093	0.073	0.0753	0.1452
2013	0.1173	0.1992	0.1963	0.0804	0.113	0.075	0.0747	0.1441
2014	0.1097	0.1871	0.1292	0.0964	0.1299	0.0916	0.0884	0.1676

The regional equipment profiles demonstrate the average localisation of the equipment types according to the self-governing regions, as shown in Table 4.

**Table 4:** Regional equipment profiles

Indicator	BC	BL	KI	NI	PV	TC	TA	ZI
average row profile	0.119	0.2458	0.1658	0.0894	0.1023	0.0649	0.0672	0.1457
equipment to population ratio	0.1246	0.2118	0.1756	0.0875	0.1107	0.0719	0.0647	0.1533
equipment density	0.9551	1.1605	0.9442	1.0217	0.9241	0.9026	1.0386	0.9504

The equipment average row profile shown in Table 4 reveals quite large regional disparities in this field in the Slovak Republic. For instance, the highest average row profile belongs to the BL Region, where its value reaches 0.1871, whereas the lowest one is associated with the TA Region at 0.884, which is less than half of the previous number. These values represent the share of the entire amount of equipment in the Slovak Republic according to localisation in the self-governing regions. They express the average share of all the equipment types in the individual self-governing regions.

The equipment to population ratio represents the amount of medical equipment per 1000 inhabitants. The highest figure belongs to the BL Region, with a value of 0.2118, whereas the lowest ratio, at 0.0647, is assigned to the TA Region. This indicates that every thousand inhabitants are served by less than one-third of the medical equipment available in the best-equipped region.

Equipment density is calculated as the ratio of the average row profile and the equipment to population ratio. It expresses the proportion of whether a share of the equipment localised in a particular region is higher than the ratio of all the medical equipment to the population of the given region. The highest value, 1.1605, is reached by the BL Region, whereas the lowest value, 0.9026, belongs to the TC Region. This finding reveals that the most-equipped region also has the best density of medical equipment.

One of the most frequently applied methods to determine how much information is available in the examined data is to use the eigenvalue with its attributes as the indicator [30]. For this purpose, we have quantified the eigenvalues of the dimensions according to the correspondence analysis, as shown in Table 5.

**Table 5:** Eigenvalues of the dimensions of the medical equipment distribution

Dimension	Eigenvalue	Percentage of variance	Cumulative percentage of variance
1	0.0230	39.75%	39.75%
2	0.0121	20.85%	60.61%
3	0.0098	16.98%	77.59%
4	0.0044	7.62%	85.20%
5	0.0040	6.85%	92.06%
6	0.0029	4.98%	97.04%
7	0.0017	2.96%	100%
8	2.40. 10^−33^	4.14. 10^−30^%	100%

According to the output of the executed correspondence analysis as seen in Table 5, there are eight dimensions, but the last one has a share of only 4.14 10^−30^%, which can be considered equal to 0. These eight dimensions represent the individual self-governing regions of the Slovak Republic. The main dimension explains 39.75% of the data inertia. This could be presented as the most representative self-governing region. The second and third dimensions add approximately the same share of inertia −20.85% and 16.98%, respectively. The fourth dimension and the successive dimensions individually produce less than a half of the inertia produced by the previous dimension. The fourth one explains 7.62%, which is only 44.84% of the inertia clarified by the previous dimension. Because the first three dimensions provide a 77.59% share of the data inertia together, we can set the number of statistically significant dimensions to three [31]. It means the three strongest self-governing regions – in terms of the strength from an angle of view of this analysis – are able to perform as the whole dataset. The rest of the analysis is concerned with this setting.

The situation of the localisation of health facilities is varied in the Slovak Republic. To measure regional disparities, the similarity of the equipment row profiles is used. We compute similarity as the chi-square distance. The output, which reflects the chi-square distances from the end of the explored period subtracted from the beginning of the explored period, is shown in Table 6.

**Table 6:** Subtraction of chi-square distances of equipment profiles between 2014 and 2008

	1	2	3	4	5	6	7	8	9	10	11	12	13	14	15	16	17	18	19	20	21	22	23	24	25	26	27	28	29
1	0	0.474	0.18	0.188	1.1	0.022	0.105	0.085	0.083	0.198	0.006	0.124	0	0.194	0.138	0.055	0.059	0.157	0.018	0.098	0.079	0.012	1.455	0.085	0.038	0.261	0.082	0.733	0.059
2	0.474	0	0.133	0.701	0.617	0.806	0.534	0.99	0.592	0.293	0.636	0.269	0.805	0.433	0.772	0.846	0.412	0.701	0.061	0.218	0.638	0.13	0.878	0.455	0.498	0.434	0.305	1.055	0.747
3	0.18	0.133	0	0.187	1.65	0.014	0.062	0.093	0.081	0.114	0.098	0.095	0.085	0.171	0.216	0.078	0.21	0.333	0.105	0.207	0.037	0.01	1.242	0.005	0.074	0.1	0.038	0.544	0.133
4	0.188	0.701	0.187	0	0.918	0.108	0.11	0.06	0.034	0.565	0.001	0.488	0.096	0.188	0.17	0.163	0.106	0.201	0.114	0.445	0.041	0.121	0.566	0.044	0.041	0.228	0.087	0.145	0.018
5	1.1	0.617	1.65	0.918	0	1.064	1.299	1.03	1.217	0.364	0.78	1.125	1.107	0.029	0.44	0.717	0.796	0.325	0.81	1.353	0.788	0.849	0.36	1.165	1.057	1.555	0.721	0.233	1.203
6	0.022	0.806	0.014	0.108	1.064	0	0.041	0.031	0.032	0.072	0.008	0.246	0.009	0.072	0.101	0.003	0.053	0.094	0.28	0.231	0.011	0.065	1.244	0.019	0.022	0.409	0.024	0.568	0.021
7	0.105	0.534	0.062	0.11	1.299	0.041	0	0.139	0.11	0.259	0.095	0.232	0.073	0.003	0.076	0.038	0.088	0.091	0.16	0.548	0.101	0.026	1.096	0.053	0.054	0.534	0.054	0.247	0.195
8	0.085	0.99	0.093	0.06	1.03	0.031	0.139	0	0.067	0.292	0.046	0.521	0.003	0.226	0.035	0.051	0.021	0.026	0.144	0.076	0.008	0.156	0.832	0.002	0.059	0.357	0.051	0.09	0.032
9	0.083	0.592	0.081	0.034	1.217	0.032	0.11	0.067	0	0.31	0.005	0.329	0.024	0.094	0.207	0.096	0.084	0.109	0.162	0.254	0.015	0.079	0.913	0.01	0.019	0.483	0.001	0.381	0.019
10	0.198	0.293	0.114	0.565	0.364	0.072	0.259	0.292	0.31	0	0.027	0.347	0.241	0.03	0.123	0.061	0.227	0.345	0.641	0.571	0.326	0.263	1.278	0.186	0.354	0.057	0.162	1.267	0.136
11	0.006	0.636	0.098	0.001	0.78	0.008	0.095	0.046	0.005	0.027	0	0.264	0.051	0.102	0.019	0.008	0.06	0.03	0.091	0.348	0.048	0.017	1.192	0.043	0.025	0.636	0.085	0.431	0.055
12	0.124	0.269	0.095	0.488	1.125	0.246	0.232	0.521	0.329	0.347	0.264	0	0.396	0.07	0.278	0.11	0.16	0.144	0.355	0.172	0.188	0.146	1.179	0.22	0.308	0.048	0.187	0.806	0.098
13	0	0.805	0.085	0.096	1.107	0.009	0.073	0.003	0.024	0.241	0.051	0.396	0	0.076	0.106	0.021	0.105	0.017	0.275	0.222	0.003	0.085	0.994	0.017	0.061	0.395	0.008	0.5	0.019
14	0.194	0.433	0.171	0.188	0.029	0.072	0.003	0.226	0.094	0.03	0.102	0.07	0.076	0	0.151	0.003	0.067	0.183	0.27	0.352	0.028	0.139	1.001	0.026	0.026	0.236	0.017	0.838	0.122
15	0.138	0.772	0.216	0.17	0.44	0.101	0.076	0.035	0.207	0.123	0.019	0.278	0.106	0.151	0	0.138	0.042	0.2	0.375	0.094	0.069	0.15	1.407	0.148	0.175	0.674	0.034	0.674	0.114
16	0.055	0.846	0.078	0.163	0.717	0.003	0.038	0.051	0.096	0.061	0.008	0.11	0.021	0.003	0.138	0	0.043	0.277	0.29	0.037	0	0.113	1.282	0.015	0.067	0.664	0.046	0.687	0.013
17	0.059	0.412	0.21	0.106	0.796	0.053	0.088	0.021	0.084	0.227	0.06	0.16	0.105	0.067	0.042	0.043	0	0.083	0.077	0.299	0.058	0.014	0.975	0.111	0.023	0.622	0.078	0.39	0.159
18	0.157	0.701	0.333	0.201	0.325	0.094	0.091	0.026	0.109	0.345	0.03	0.144	0.017	0.183	0.2	0.277	0.083	0	0.167	0.574	0.023	0.093	1.092	0.077	0.013	0.399	0.009	0.654	0.08
19	0.018	0.061	0.105	0.114	0.81	0.28	0.16	0.144	0.162	0.641	0.091	0.355	0.275	0.27	0.375	0.29	0.077	0.167	0	0.289	0.197	0.088	0.067	0.219	0.218	0.252	0.229	0.208	0.01
20	0.098	0.218	0.207	0.445	1.353	0.231	0.548	0.076	0.254	0.571	0.348	0.172	0.222	0.352	0.094	0.037	0.299	0.574	0.289	0	0.23	0.153	0.773	0.331	0.378	0.899	0.281	0.111	0.158
21	0.079	0.638	0.037	0.041	0.788	0.011	0.101	0.008	0.015	0.326	0.048	0.188	0.003	0.028	0.069	0	0.058	0.023	0.197	0.23	0	0.105	1.017	0.048	0.045	0.361	0.015	0.497	0.064
22	0.012	0.13	0.01	0.121	0.849	0.065	0.026	0.156	0.079	0.263	0.017	0.146	0.085	0.139	0.15	0.113	0.014	0.093	0.088	0.153	0.105	0	0.81	0.033	0.084	0.382	0.024	0.518	0.003
23	1.455	0.878	1.242	0.566	0.36	1.244	1.096	0.832	0.913	1.278	1.192	1.179	0.994	1.001	1.407	1.282	0.975	1.092	0.067	0.773	1.017	0.81	0	0.968	1.046	0.693	0.72	0.153	1.546
24	0.085	0.455	0.005	0.044	1.165	0.019	0.053	0.002	0.01	0.186	0.043	0.22	0.017	0.026	0.148	0.015	0.111	0.077	0.219	0.331	0.048	0.033	0.968	0	0.027	0.37	0.006	0.421	0.144
25	0.038	0.498	0.074	0.041	1.057	0.022	0.054	0.059	0.019	0.354	0.025	0.308	0.061	0.026	0.175	0.067	0.023	0.013	0.218	0.378	0.045	0.084	1.046	0.027	0	0.548	0.029	0.519	0.154
26	0.261	0.434	0.1	0.228	1.555	0.409	0.534	0.357	0.483	0.057	0.636	0.048	0.395	0.236	0.674	0.664	0.622	0.399	0.252	0.899	0.361	0.382	0.693	0.37	0.548	0	0.323	0.125	0.487
27	0.082	0.305	0.038	0.087	0.721	0.024	0.054	0.051	0.001	0.162	0.085	0.187	0.008	0.017	0.034	0.046	0.078	0.009	0.229	0.281	0.015	0.024	0.72	0.006	0.029	0.323	0	0.531	0.14
28	0.733	1.055	0.544	0.145	0.233	0.568	0.247	0.09	0.381	1.267	0.431	0.806	0.5	0.838	0.674	0.687	0.39	0.654	0.208	0.111	0.497	0.518	0.153	0.421	0.519	0.125	0.531	0	0.441
29	0.059	0.747	0.133	0.018	1.203	0.021	0.195	0.032	0.019	0.136	0.055	0.098	0.019	0.122	0.114	0.013	0.159	0.08	0.01	0.158	0.064	0.003	1.546	0.144	0.154	0.487	0.14	0.441	0

Legend: figures written in the upper right half of the matrix belong to year 2008, and numbers displayed in the lower left half of the matrix belong to year 2014; main diagonal is the same for both years because of its characteristic; figures written in black are positive numbers, and numbers written in red are negative numbers

If we compare individual distances between the equipment types at the beginning and at the end of the observed period, we can observe changes in the similarities of the same equipment type pairs. There are two cases in which no change occurred after the analysed period: between the angiograph and the group of colonoscopes, sigmoidoscopes and proctoscopes and between the laryngoscope and mammograph.

**Fig. 2:**
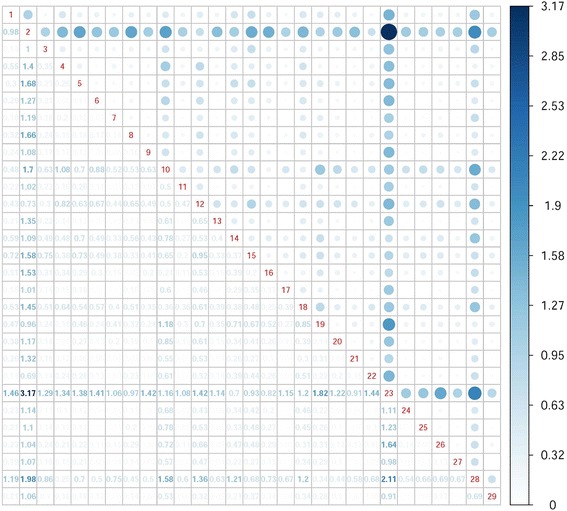
Chi-square distances of equipment row profiles

Figure 2 presents an analysis of the equipment row profiles.

**Fig. 3:**
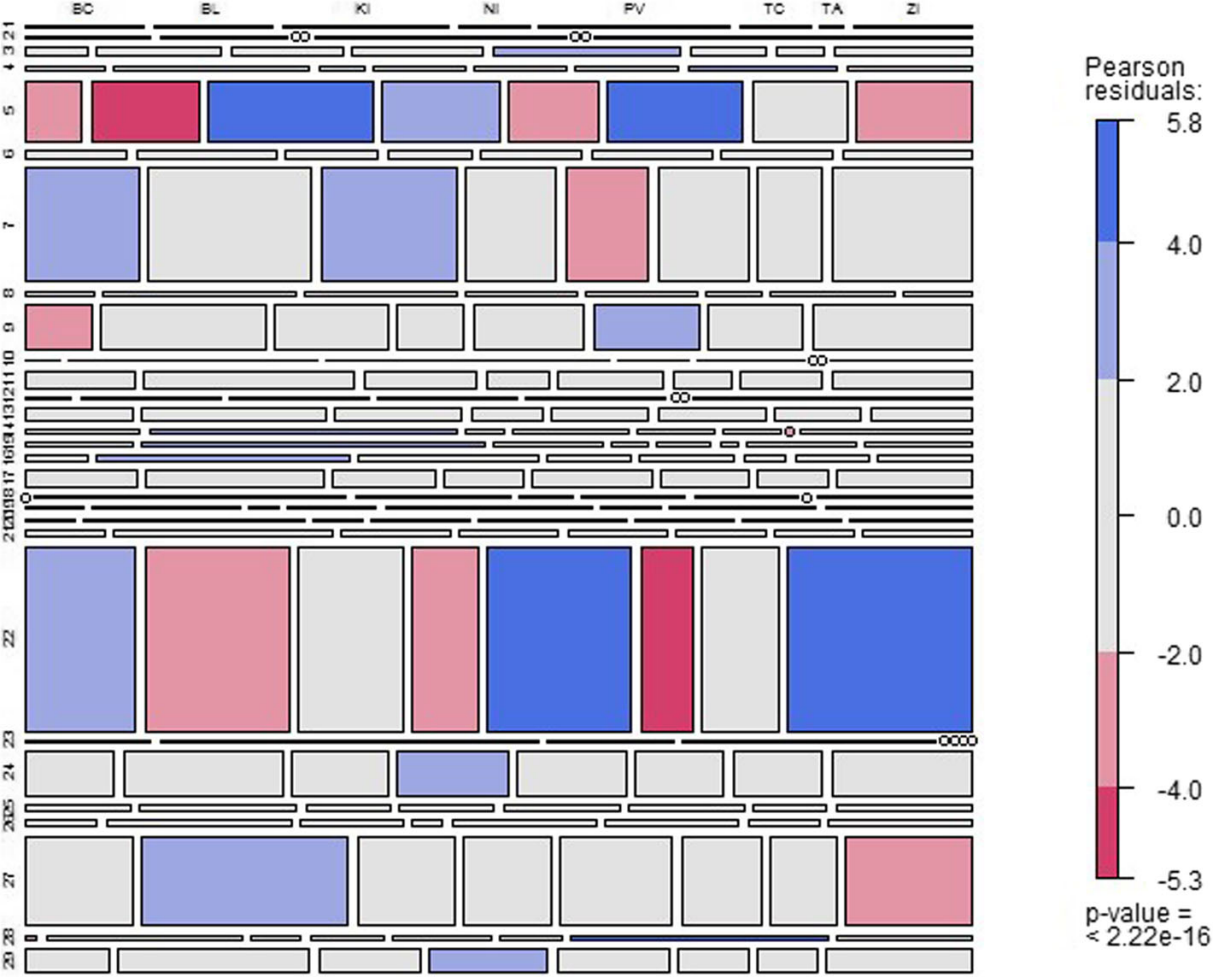
Dependence between the number of the equipment and its localisation

Figure 2 displays the chi-square distances of the equipment row profiles. If we consider this table matrix, we can observe that the matrix is symmetrical because the similarity of the equipment types based on the chi-square distance quantification is not directional or causal. Zeros in the main diagonal represent the identity of the same equipment row profiles. The smaller the distance between two row profiles is, the more similar they are. In our case, this indicates that the lower similarity values are between the two equipment row profiles. The most analogous localisation of these two equipment types is in the Slovak Republic. The largest distance can be seen between the brachytherapy apparatus and the positron tomograph, which indicates that the localisation of equipment for brachytherapy and the positron tomograph is the most different with a chi-square distance of 3.17. This is followed by the pair of the uretroscope and brachytherapy apparatus, which are distant from each other by 1.98. Generally, brachytherapy apparatus is the most distant one from all the equipment because as the only one device it is not nearer to any remaining equipment than distance of 1 is. Dependence between a number of the equipment and its localisation is demonstrated in Fig. 3.

The mosaic diagram in Fig. 3 presents the localisation of the equipment shown in the y-axis types according to the separate self-governing regions presented on the x-axis in such a way that the quadrilateral area represents the share of the number of the particular type of equipment to the total number of all equipment in the entire Slovak Republic or in the particular self-governing region. Blue shading represents a situation in which the number of equipment of a particular type is higher than in the case of their uniform distribution among all the healthcare facilities in the Slovak Republic. The red shading indicates a state in which there is a smaller number of equipment of that type localised in the specific self-governing regions than in the case of uniform localisation.

These statements can be formulated into the two following hypotheses:

- H_0_: there is no dependence between the number of equipment of the same type and its localisation;
- H_1_: there is a dependence between the number of the equipment of the same type and its localisation.

**Fig. 4:**
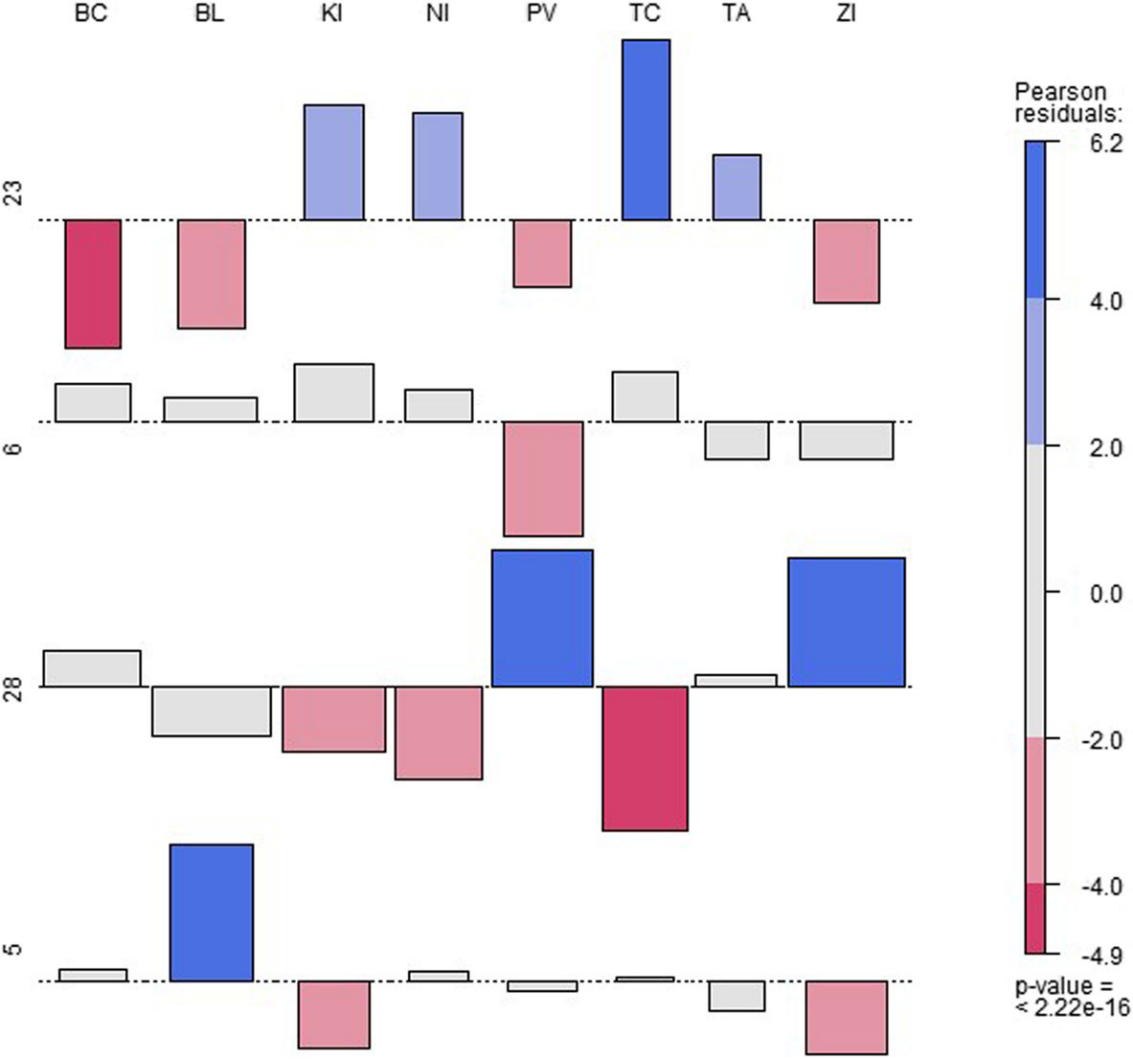
The most numerous equipment localisation

To determine which statement is statistically true, we use standardised Pearson’s residuals. Pearson’s chi-square test statistics reach a value of 532.08 at 196 degrees of freedom. The *p*-value stands at 7.82 10^−33^, which can be regarded as 0, so we do not reject the zero hypothesis H_0_. This indicates that there is no evidence that dependence between the equipment type and its localisation is present according to this analysis. The most numerous equipment localisation is demonstrated in Fig. 4.

To visualise the most numerous equipment, we have chosen four types of equipment with the highest absolute quantities: monitoring devices, electrocardiographs, ultrasound devices and dialysis monitors. The association diagram in Fig. 4 shows the relationship between the type of equipment demonstrated on the y-axis and its location region visualised on the x-axis.

The hypotheses are as follows:

- H_0_: there is no association between the number of equipment of a particular type and its localisation;
- H_1_: there is an association between the number of equipment of a particular type and its localisation.

To verify the relationship between the types of equipment and the self-governing regions, we applied Pearson’s chi-square test. The statistics reached a value of 265.30 at 21 degrees of freedom with a *p*-value of 2.2 10^−16^, which can be considered to be 0. According to this result, we do not reject the zero hypothesis H_0_, which says that there is no association between equipment type and its localisation.

Another analytical dimension is represented by causality between mortality and the localisation of the medical equipment. Our goal is to examine the relation between the health policy platform with a preference for the diagnoses of the circulatory system in the ninth chapter of the International Statistical Classification of Diseases and Related Health Problems maintained by the World Health Organization and the distribution of medical equipment throughout the Slovak regions.

The most common diagnoses causing death are the ones assigned to the circulatory system group. For the time span from 2008 to 2013, the correlation of the standardised mortality rate and the localisation of healthcare equipment according to the self-governing regions is calculated. The BL Region is the only one that shows a very weak correlation at the level of −0.1280. The TC Region and the TA Region are characterised by a low positive correlation reaching 0.3891 and 0.4017, respectively, whereas the NR Region is represented by a low negative correlation at −0.3787. The remaining four regions demonstrate cases with high correlations. The PV Region and the BC Region have a high positive correlation at 0.6209 and 0.8310, respectively, whereas the ZI Region and the KI Region have a high negative correlation at −0.7976 and −0.8615, respectively. A low correlation indicates that there is no association between the amount of healthcare equipment and the decreasing standardised mortality rate. The most desirable occurrence happens when the increasing number of healthcare equipment helps to lower the standardised mortality rate.

## Discussion

The medical equipment in the health facilities all over the Slovak Republic is localised very unproportionally. Its distribution does not follow the optimal localisation according to the map of the health facilities.

Several health facilities have limited access to tomography or magnetic resonance imaging devices. Situations may occur in which a patient in need must wait several months to be examined by x-ray computed tomograph. The origin of this situation is debatable. The most probable cause is the absence of financial resources that should be provided by health insurance companies. Currently, the Slovak economy and the related health system are established in a way that does not allow the natural reproduction of health technologies. Healthcare providers are not able to gather the financial resources to purchase new equipment. Therefore, it is necessary to address this issue and to communicate it at the government level. The need to acquire diagnostic equipment to coordinate public health departments is also an important issue. One solution could be the initiation of an adequate norm that would force healthcare providers to renovate medical equipment in the corresponding time period. An alternative to resolve this problem would be to introduce the process of financing the acquired health equipment from the state budget or by reimbursement from the health insurance companies. For instance, investments in radiological technology should be one of the most important indicators monitored within the strategic framework of the Slovak health service. Another issue is the inappropriate distribution of medical equipment throughout the Slovak regions, which have significant regional disparities between healthcare providers. This issue is closely tied to the autonomy of the self-governing regions of the Slovak Republic because they are also responsible for the management of healthcare providers supported by the state budget.

Many research teams have analysed medical equipment and the quantification of its causalities. Similarly, many scientific studies have focused on the status of medical equipment as part of a country’s informatisation process in the context of various reforms or in research on regional disparities in terms of health [32–35]. Other authors focus on selected aspects of the health system and examine the influence of medical equipment on economic categories, the availability of healthcare and its real impact on regions [36–42]. Several studies that have conducted research on the geographical distribution of medical equipment and its causalities are heterogeneous in terms of the research targets as well as their methodological mechanisms that significantly limit international comparison. There are visible the considerable different results than obtained from this analysis. The main evidence may be provided by a study that analyses medical equipment in Japan. The paradox is that even in Japan, which has the highest number of computed tomographs and magnetic resonance imaging devices per inhabitant in the world, the geographic distribution of these technologies is currently unknown. Moreover, nothing is known regarding the cause and effect relationship between the number of diagnostic devices and their geographic distribution [43]. These facts create a wide platform for subsequent studies, whose findings are necessary for various types of policies in individual countries. The main strength of the study lies in a combination of an investigation that has not been yet done and usage of the new modern type of analysis. There is a lack of such studies in the Slovak Republic. The potential weakness is hide in a short period that is observed in the analysis. This is due the fact that there had been no such data before the beginning of the explored period.

## Conclusion

Global ageing is a process that is related to the development of the standardised mortality rate. These two factors have a significant impact on the health system of a country and are influenced by a number of socio-economic determinants. Moreover, several aspects influence the processes discussed here. For instance, the health insurance system, with its excessive expenses that are intended for the entire network of healthcare providers, plays an important role in the quality of treatment processes and the sophistication of medical technology. Worldwide, the historical development of scientific and technical progress and the rate of implementation of research into practice is diverse. The Slovak Republic lacks the ability for long-term monitoring of the status of medical equipment in health facilities. Some issues influence healthcare and associated treatment.

The aim of this study is to examine the localisation of selected parameters in use of medical equipment in the Slovak Republic and subsequently to discover potential regional disparities. This aim was partially fulfilled by the finding of unproportional localisation of the medical equipment, although there is to note that further research is needed in this field. The results demonstrate regional disparities in the use of medical equipment throughout the whole analysed period. There are also the regions where medical equipment is not present or is present in considerable low numbers, which creates an inefficient situation. The substantial meaning of the study is to reveal the potential regional disparities throughout the various types of the analyses.

There is a lot of possibilities how to conduct this research in future. The most important point is not created by the obtained results from the conducted analysis. These findings should serve as a monitoring platform to evaluate the accessibility and quality of healthcare technologies for use in this country. It is necessary to obtain more detailed data to obtain more valuable conclusions from this analysis. The results can serve to policymakers and they can create a substantial part of the potential future support systems. We will continue our current research activities and cooperate with the related institutions of the Slovak health system.
